# Editorial: Mental health economics and public mental health policy: mental health services costs, quality and its impact on reducing the burden of mental illness

**DOI:** 10.3389/frhs.2023.1267580

**Published:** 2023-08-23

**Authors:** Denise Razzouk

**Affiliations:** Faculty of Medicine, Universidade Metropolitana de Santos, Santos, Brazil

**Keywords:** mental health services, mental health economics, burden of mental disorders, quality of mental health care, costs of mental disorders, mental health policy, return to investment

**Editorial on the Research Topic** Mental health economics and public mental health policy: mental health services costs, quality and its impact on reducing the burden of mental illness

## Introduction

Mental Health Economics has a crucial role to guide decision-makers and planners in evaluating the implementation and the costs and benefits of health policies, services and interventions. The majority of the literature available is usually focused on the costs of intervention and services regardless of the importance of measuring the quality of mental healthcare after the implementation of new therapeutics and services (1). Cost-effectiveness studies are important tools for comparing costs and benefits among therapeutical interventions but are hardly applicable to all contexts and their results vary according to multiple factors not always measurable. For instance, the implementation of a cost-effective public health policy might result in an opposite outcome than is expected if its implementation isn't adequately evaluated and monitored.

In this Research Topic, we have four manuscripts discussing different aspects related to national investments in mental healthcare in low and middle-income countries, costs of mental health services in the real-world scenario, the implementation challenges and guidelines of new digital health technologies in mental health services and the critical issues of evaluating mental health services costs without mental health outcomes data.

## National investments in mental healthcare in low and middle-income countries

Chisholm et al. estimated the return on investment and the cost-benefit ratios of mental health investments in seven Asian and African countries demonstrating the remarkable economic and social return to society of scaling up a modest amount of investment in mental health interventions considering different contexts, GDP and countries characteristics. Of note, the intrinsic value of mental health was taken into account, which is a crucial point when policymakers only base their decisions on narrow perspectives used in the cost-effectiveness and clinical trial studies dismissing the societal and economic burden of mental illness. In this study, the economic burden was up to 1% of countries’ GDP while the need for scaling-up investment would be less than 0.14% of countries' GDP and with a benefit-cost ratio for depression up to 30:1.

## Mental healthcare costs

de Oliveira et al. analysed an administrative database of mental healthcare expenditures and explored the characteristics of patients with chronic psychosis associated with these costs across one decade in Canada. As expected, hospitalisation was the main part of the total costs, but the interesting finding is that hospitalisations increased over time, especially in the presence of other medical comorbidities (>5), highlighting the importance of integrating and monitoring physical and mental care to optimise costs and outcomes. Of note, the distribution of mental health expenditures varied over time with a remarkable increase in acute medical hospitalisations, outpatient visits, medication and home care. These findings shed light on the need of measuring costs in a comprehensive approach because costs do not necessarily decrease over time with the decrease in psychiatric hospitalisations.

## Mental health care quality and technology implementation

The evaluation of mental health services quality and health policy investments depends on the quality of data. Of note, outcomes in mental health should be measured in a comprehensive approach considering not only symptom improvement but social, occupational and functional recovery. However, administrative health systems rarely contain relevant or accurate data regarding mental health outcomes. Mental health outcomes and costs vary over time, context and type of service and disorder. For this reason, a longitudinal analysis is crucial to assess the value of investments, services costs, planning health policies and improving mental health services.

Mark described the challenges and gaps in evaluating and planning mental health services using data from USA's mental health system database. The need for a linkage between databases regarding outcomes, costs and services delivered remains a crucial challenge to evaluate the quality of mental health services. The administrative databases have several limitations in terms of evaluating costs and outcomes. In this manuscript, Mark purposed some measures for mental health information system improvement, focusing on outcomes data.

Iorfino et al. discussed the potential positive impacts of new digital health technologies for mental healthcare quality and efficiency targeting young people using dynamic simulation modelling and describing a framework on how it was implemented in health services research in Australia. The authors described some measures needed to guide the implementation of such tools in the real world. Of note, the implementation of new health technologies requires the participation of health services, funders, managers and also patients. Monitoring the implementation process of such tools is crucial for further evaluations of mental health services' efficiency, cost-effectiveness and quality of care.

## Conclusion

The social and economic impact of mental health on society has been growing over the decades and several measures have been purposed to minimise the burden of mental illness. A myriad of solutions including preventive measures, medication, psychological therapies, social therapies and the advent of new health technologies promise better outcomes and social and economic returns. However, there are, at least, three major bottlenecks for promoting mental health and enhancing mental health system efficiency: the lack of effective investment; poor quality of mental health system data and a paucity of rigour evaluations for the implementation and for the evaluation of implemented services and policy.
